# Synergy between Antimicrobial Peptides and Lipid Nanoparticles for Skin Infection Control

**DOI:** 10.1021/acsami.5c18033

**Published:** 2025-11-29

**Authors:** Lucas de Alcântara Sica de Toledo, Hélen Cássia Rosseto, Danieli Fernanda Buccini, Octávio Luiz Franco

**Affiliations:** † S-Inova Biotech, Programa de Pós-Graduação em Biotecnologia, 186072 Pontifícia Universidade Católica Dom Bosco, Campo Grande, MS 79117900, Brazil; ‡ Faculty of Pharmaceutical Sciences, Food and Nutrition, 54534 Universidade Federal de Mato Grosso do Sul, Campo Grande, MS 79070900, Brazil; § Centro de Análises Proteômicas e Bioquímicas, Pós-Graduação em Ciências Genômicas e Biotecnologia, Pontifícia Universidade Católica de Brasília, Brasília, DF 70790160, Brazil

**Keywords:** antimicrobial peptides, lipid nanoparticles, controlled drug release, multidrug-resistant bacteria, skin infections, nanomedicine

## Abstract

Skin infections caused by multidrug-resistant bacteria are an increasing public health concern, requiring therapeutic approaches. Antimicrobial peptides (AMPs) have been studied as alternatives to conventional antibiotics due to their broad-spectrum activity and lower risk of inducing resistance. However, their clinical application remains limited due to enzymatic instability, rapid clearance, and potential toxicity. Lipid nanoparticles (LNPs) have emerged as a promising strategy to protect and deliver AMPs in a controlled manner, enhancing their efficacy while reducing side effects. This review explores how AMP encapsulation within LNPs can improve stability, prolong antimicrobial activity, and optimize skin penetration. Despite recent advances, challenges, such as scalability, regulatory approval, and clinical application, must still be addressed to bring this approach into widespread use for skin infection treatment. This review explores the synergy between AMPs and LNPs, focusing on their mechanisms of action, *in vitro* and *in vivo* efficacy, and controlled drug release strategies. We highlight the advantages of LNP encapsulation, including increased bioavailability, prolonged antimicrobial activity, and reduced systemic toxicity. Various LNP types, such as solid lipid nanoparticles (SLN) and nanostructured lipid carriers (NLC), are discussed in relation to their structural properties and impact on AMP delivery. Despite these advancements, challenges remain in optimizing formulation scalability, regulatory approval, and clinical translation. Future research should focus on hybrid delivery platforms, stimuli-responsive systems, and large-scale production techniques to enhance AMP-based nanotherapies. Integrating LNPs into AMP therapeutics represents a transformative approach to combating antibiotic resistance and improving skin infection management.

## Graphical abstract

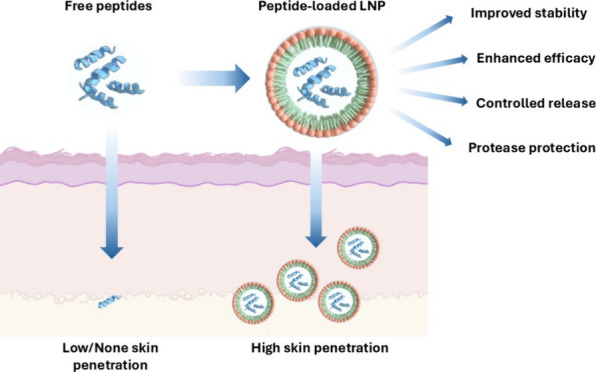

## 1 Introduction

Skin infections, alongside inflammatory disorders, including acne, eczema, and chronic wounds, affect millions of people worldwide. In addition to compromising patients’ quality of life, treating these conditions has become increasingly challenging due to the rise of antibiotic-resistant bacteria, notably the clinically critical strains like methicillin-resistant *Staphylococcus aureus* and *Acinetobacter baumannii*.^1^ The overuse and, in many cases, indiscriminate prescription of antibiotics have accelerated the emergence of multidrug-resistant strains, leading to prolonged infections and rising healthcare costs.^2^ Given this scenario, new therapeutic strategies are essential to combat bacterial resistance while ensuring effective and safe treatments.

Antimicrobial peptides (AMPs) represent a groundbreaking approach to combating resistant pathogens, offering mechanisms of action that reduce the likelihood of resistance development. They have emerged as a promising alternative in combating resistant bacteria due to their broad-spectrum activity and unique mechanisms that disrupt microbial membranes, reducing the likelihood of resistance development.^3–6^

Despite their potential, AMPs face critical limitations on their application *in vivo*, which remains challenging, as they often show limited stability and bioavailability, susceptibility to proteolytic degradation, rapid excretion, and may exhibit toxicity at higher concentrations, mainly when used topically on sensitive skin.^7–11^ These limitations underscore the importance of developing innovative nanotechnology-based delivery systems, such as lipid nanoparticles (LNPs), to protect AMPs from enzymatic breakdown while ensuring targeted and sustained release at the site of infection; hence, LNPs would play a transformative role.^3,10–14^ By overcoming these barriers, AMPs could emerge as powerful alternatives or complements to conventional antibiotics in the fight against antimicrobial resistance.^3,10,11^

Encapsulating AMPs within LNPs has proven to be an effective solution to overcome challenges such as enzymatic degradation and toxicity at high concentrations. This controlled-release system protects antimicrobial peptides, extends their activity in the body, and enhances their penetration into deeper skin layers. Thus, combining AMPs with nanocarriers significantly expands their therapeutic potential, making them a viable alternative to conventional antibiotics.^15–18^

For example, LNPs are an effective solution for entrapping AMPs, protecting them against enzymatic degradation and enabling controlled release at the injection site. This approach not only enhances the stability of AMPs but also improves their bioavailability and therapeutic efficacy.^19,20^

The combination of AMPs and LNPs not only addresses these limitations but also enhances the therapeutic potential of AMPs by providing stability, controlled release, and targeted delivery. This synergy between biologically active peptides and advanced delivery systems bridges the gap between therapeutic innovation and clinical practicality.^11,21,22^

Lipid nanoparticles have revolutionized skin infection treatments by offering a more efficient and targeted approach. Due to their biocompatibility and ability to penetrate the skin barrier, they facilitate the delivery of antimicrobial agents directly to the site of infection. Beyond improving drug stability and absorption, LNPs also minimize systemic side effects, which is a crucial advantage in the treatment of localized infections and inflammatory skin conditions. This technology opens new possibilities for safer and more effective therapies.^23,24^ This innovation has opened new avenues for addressing the limitations of existing treatments, particularly for complex cases involving chronic wounds or multidrug-resistant pathogens.

Furthermore, the skin’s unique structure presents both challenges and opportunities for topical drug delivery. The stratum corneum, the outermost layer of the epidermis, acts as a formidable barrier. It is crucial to address the efficiency with which LNPs can reach deeper skin layers.^3,16,23,25,26^

Therefore, this delivery strategy is effective for superficial infections (e.g., impetigo), folliculitis, or conditions in which the skin barrier is already compromised, such as in chronic wounds. However, for infections located in the deep dermis or subcutaneous tissue, such as cellulitis, topical LNP delivery would benefit from strategies such as combining it with physical penetration enhancers (e.g., microneedles) to reach deeper targets.^3,16,23,25^

The ability of lipid nanoparticles to overcome the stratum corneum barrier is crucial for treating localized skin infections. Indeed, *in vitro* and *ex vivo* skin permeation studies consistently demonstrate a stark contrast in delivery efficacy: while free peptides are largely retained on the skin’s surface, LNP-encapsulated AMPs achieve significantly deeper penetration into the viable epidermis and dermis. As illustrated in Figure 1, this enhanced delivery ensures that higher, therapeutically relevant concentrations of the peptide reach the site of infection, directly overcoming the primary penetration limitations associated with free AMPs.

**Figure 1:**
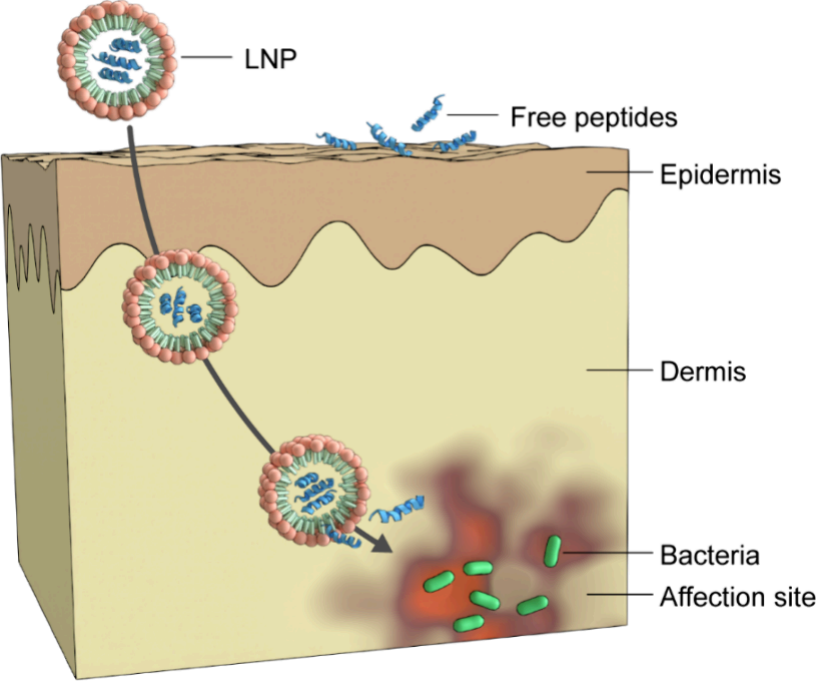
Cutaneous penetration and release of antimicrobial peptides (AMPs) freely and from lipid nanoparticles (LNPs).

Recent advances in nanotechnology have further refined the development of LNP, enabling precise control over particle size, charge, and encapsulation efficiency.^3^ These parameters are critical for optimizing the interaction of LNP with skin tissues and ensuring a consistent drug delivery. Additionally, the adaptability of LNP formulations allows for incorporating various therapeutic agents, including hydrophilic and hydrophobic compounds, broadening their applicability in dermatology and beyond.^3,12,13,16^ The synergy between AMP and LNP is exemplified by LL-37 and serpin A1 encapsulated in SLNs. These peptide-loaded nanoparticles demonstrated accelerated wound healing and potent antibacterial activity through controlled and sustained release, aligning innovation with clinical efficacy.^21,22^

To effectively translate the therapeutic potential of AMPs into clinical reality, the choice of a delivery system is paramount. An ideal nanocarrier must excel across several key metrics: high encapsulation efficiency to ensure a sufficient payload, robust stability to protect the peptide in biological environments, a precisely controlled release profile to maintain therapeutic concentrations, and excellent biocompatibility to minimize adverse effects. While various platforms, including liposomes and polymeric nanoparticles, have been explored, lipid nanoparticles (LNPs) have consistently demonstrated a superior profile for this specific application.^14,16,27^ To critically evaluate these platforms, Table 1 provides a comparative analysis of LNPs against other common nanocarriers.

**Table 1:** Comparative Analysis of Nanocarrier Systems for Antimicrobial Peptide Delivery

nanocarrier system	entrapment efficiency (EE)	stability	release profile	cytotoxicity and biocompatibility	reference
liposomes	low to moderate (10–40%) for hydrophilic AMPs. Can be improved with specific lipid compositions or loading techniques	moderate. Prone to physical instability, such as fusion and aggregation. Peptide leakage during storage and *in vivo* is a significant concern	release is primarily diffusion-based and difficult to sustain over long periods without complex modifications	composed of phospholipids, which are highly biocompatible	^28,29^
polymeric nanoparticles	moderate to high (50–90%) Highly dependent on the polymer–peptide interaction and fabrication method	high. The solid polymeric matrix provides good protection against enzymatic degradation	often biphasic with a significant initial “burst release”, which can lead to off target toxicity, followed by a slower, diffusion-based phase	while generally considered safe, the acidic degradation of polymers like PLGA can cause local inflammation and may denature the encapsulated peptide	^30^
solid lipid nanoparticles (SLNs)	moderate to high (60–95%). Excellent for lipophilic or hydrophilic AMPs. The highly ordered crystalline structure can sometimes limit payload	very high. The solid lipid core provides superior physical stability and effectively protects AMPs from enzymes, preventing premature degradation	often sustained and prolonged. Characterized by slow, diffusion-based release from the solid matrix, ideal for chronic applications; with minimal burst effect	composed of physiological, biodegradable lipids, leading to very low cytotoxicity and high skin tolerance	^13,14,21,22^
nanostructured lipid carriers	high to very high (>80%). The imperfect, amorphous lipid matrix created by blending solid and liquid lipids increases the area for drug loading and minimizes drug expulsion	excellent. Maintains the high stability of SLNs while the less-ordered core structure enhances peptide retention and protection over time	offers more flexibility than SLNs, allowing for modulated release profiles while still avoiding significant burst release	similar to SLNs, composed of biocompatible lipids, ensuring minimal toxicity and high suitability for dermatological use	^22,31–33^

As summarized in Table 1, LNPs, and particularly NLCs, demonstrate a superior overall profile for AMP delivery compared to conventional nanocarriers. The solid lipid matrix of both SLNs and NLCs provides exceptional physical stability, effectively preventing the premature peptide leakage that often plagues liposomal formulations.^13,14,16,34^ Furthermore, LNPs offer a more reliable and sustained release mechanism, mitigating the problematic initial burst release frequently observed with polymeric systems like PLGA, which can lead to local toxicity and wasted therapeutic agent.^34,35^ Most importantly, the use of physiologically and biodegradable lipids renders LNPs highly biocompatible, avoiding the potential for inflammatory responses linked to the acidic degradation products of some polymers. These advantages, high encapsulation efficiency, superior stability, controlled release, and excellent safety profile, provide a strong rationale for focusing on LNPs as the premier platform for developing next-generation AMP-based therapies for skin infections.^13,14,16,34^

This review will explore the potential of AMP-loaded LNP as an innovative therapeutic system for treating skin conditions, exploring the mechanistic and translational synergy between these two components, especially within the dermatological context. This review is distinguished from the existing literature by providing an analysis of how the physicochemical properties of LNPs can be engineered to solve AMPs’ limitations. We will examine recent advancements in LNP technology, discuss *in vitro* and *in vivo* findings related to their efficacy and safety, and highlight future directions for optimizing these systems in clinical settings.

## 2 Antimicrobial Peptides: Challenges and Therapeutic Innovations

AMPs are naturally occurring peptide molecules that play a critical role in the innate immune response, providing broad-spectrum activity against bacteria, fungi, and viruses. They can be categorized into two primary groups based on their synthesis pathway: ribosomal AMPs, such as bacteriocins, which are directly synthesized from mRNA, and nonribosomal AMPs, produced by enzymatic complexes. This classification reflects the diversity of their structure and functional mechanisms, making them versatile tools in combating microbial resistance.^36^ AMPs may have different mechanisms of action, such as the formation of pores in the bacterial membrane, inhibition of cell wall synthesis, and interaction with specific bacterial receptors.^4,37^ For example, nisin, a widely studied bacteriocin, acts by inhibiting peptidoglycan synthesis, while others, such as cerein 7B and cerein B4080, form pores in bacterial membranes, causing cell lysis.^38,39^ These properties make bacteriocins effective in treating infections caused by *Staphylococcus aureus*, including methicillin-resistant strains (MRSA), which are often associated with skin infections.^38^

In addition to bacteriocins, other AMPs, such as LL-37, and synthetic peptides, such as WR12 and D-IK8, demonstrate significant efficacy against skin pathogens, notably due to their immunomodulatory and wound-healing properties.^4,36^ These peptides not only eliminate pathogenic microorganisms but also modulate the host’s immune response, favoring the recruitment of immune cells and promoting tissue regeneration.^36^ Another crucial mechanism of AMPs is bacterial biofilm disruption. The ability of AMPs to penetrate and disrupt biofilms increases the efficacy of antimicrobial therapies, significantly reducing the bacterial load at sites of infection.^36,40^

One of the main obstacles in treating chronic infections is the formation of bacterial biofilms, which act as a protective shield against traditional antibiotics. Some AMPs, like LL-37, have shown the ability to break through these structures, significantly reducing the bacterial presence and improving treatment effectiveness. This makes them especially valuable in cases where standard antibiotics fail to deliver results.^40^

Despite their therapeutic potential, AMPs face challenges related to stability and delivery in biological environments.^41^ Bacteriocins and other AMPs are susceptible to protease degradation and face physical barriers, such as the stratum corneum, which limits their penetration into the deeper layers of the skin.^41,42^ To overcome these limitations, controlled release technologies, such as LNPs, have been explored.^21,22^ These nanoparticles protect AMPs from enzymatic cleavage, improve their bioavailability, and allow a localized and modified release, alleviating side effects and increasing therapeutic efficacy.^21,22,43^ Recent studies indicate that AMPs encapsulated in LNPs present greater openness and prolonged activity, making this approach a promising strategy for treating skin infections caused by multidrug-resistant pathogens.^44^

Integrating AMPs into LNPs offers a promising solution to their limitations. Encapsulation within LNPs protects AMPs from enzymatic degradation, enhances their stability, and facilitates their controlled release, ensuring prolonged therapeutic activity. Furthermore, this approach reduces toxicity by targeting AMPs directly to infected sites, minimizing systemic exposure. These advancements position LNPs as a transformative platform for the therapeutic application of AMPs, especially in combating multidrug-resistant pathogens.^43,44^

AMPs represent a vital component in the fight against antimicrobial resistance, offering diverse mechanisms of action and significant therapeutic potential. However, their clinical translation requires overcoming key challenges related to stability and delivery. Leveraging nanotechnology, particularly LNPs, can address these challenges, opening new horizons for treating resistant infections and chronic skin conditions.

## 3 Lipid Nanoparticles as Drug Carriers

LNPs are emerging as highly promising drug delivery systems due to their ability to encapsulate different types of therapeutic agents while maintaining biocompatibility and biodegradability. These nanoparticles are composed of a combination of solid lipids, surfactants, and cosurfactants, which help stabilize the drug and improve its absorption by the body.^12,13^ In dermatological applications, LNP are especially promising, as they can deliver drugs directly to the affected skin regions, minimizing systemic exposure and potential side effects.^32,45^

When used as carriers for AMPs, LNPs address several critical limitations associated with AMP, including susceptibility to enzymatic degradation and potential cytotoxicity at higher concentrations. Encapsulation within LNP provides a protective barrier, ensuring sustained and localized release, which maintains therapeutic concentrations over time.^17,18^ Additionally, LNP facilitates deeper skin penetration by integrating with lipid layers in the stratum corneum, thereby improving treatment efficacy without compromising the skin’s natural barrier.^33^

Research into LNP as a drug carrier has shown promising results across various skin conditions. Studies report that LNP formulations containing AMP can effectively target bacterial biofilms and multidrug-resistant strains, which are common challenges in chronic wound care and dermatological infections.^15,17^ However, despite their potential, challenges remain in optimizing LNP for clinical use, including ensuring consistent drug loading, preventing peptide aggregation, and establishing large-scale production methods that maintain particle stability and reproducibility.^34,46^ For example, the peptide LL37 was entrapped into NLCs, showing a gain in its therapeutic application, reducing side effects, and protecting the AMP against degradation.^22^

In summary, the LNP represents a highly versatile platform for AMP delivery in dermatology, offering solutions to critical obstacles associated with AMP use. This section will explore different LNP types, their controlled release mechanisms, and recent *in vitro* and *in vivo* findings regarding their safety, efficacy, and application in treating skin infections.

Lipid nanoparticles are classified into several categories based on their composition and structure. The most prominent types include solid lipid nanoparticles (SLN), nanostructured lipid carriers (NLC), and lipid-polymer hybrid nanoparticles. SLNs are among the first developed lipid-based nanocarriers consisting of solid lipids at room and physiological temperatures. This solid lipid core provides a stable matrix for encapsulating hydrophobic drugs, making SLNs particularly suitable for enhancing the solubility and stability of poorly water-soluble compounds. The solid lipid structure also contributes to the sustained release of encapsulated drugs, extending their therapeutic activity and reducing dosing frequency.^12,13^ Despite their advantages, SLN formulations have certain limitations. The rigid crystalline structure of solid lipids can limit drug loading capacity, as many drugs may not easily integrate into the tightly packed lipid matrix. Moreover, the crystalline lipid matrix can undergo polymorphic transitions during storage, potentially leading to drug expulsion. Such issues necessitate careful selection of lipid types and optimization of processing parameters to ensure consistent drug retention and release properties. Recent advancements, including incorporating functional excipients and using advanced preparation techniques, have shown promise in addressing these challenges, thereby enhancing the performance of SLN in dermatological and systemic applications.^47,48^

Another type of LNP is NLC, which represents a second-generation lipid nanoparticle system designed to overcome the limitations of SLNs. These carriers are formulated using a combination of solid and liquid lipids, resulting in an amorphous or less-ordered lipid matrix. This structure prevents the tight crystallization seen in SLNs, thereby allowing for increased drug loading capacity and reducing the risk of drug expulsion during storage.^47^

The flexibility of NLC formulations makes them particularly advantageous for dermatological applications. Their lipid matrix can be tailored to encapsulate various therapeutic agents, including hydrophilic and lipophilic drugs. Moreover, including liquid lipids enhances the nanoparticles’ ability to integrate with skin lipids, improving drug penetration into deeper skin layers. For AMP, NLCs have demonstrated significant potential in modifying the release profile and maintaining peptide stability, for example, the LL37, which is critical for managing chronic infections and inflammation.^22,49^

In addition to their functional advantages, NLC can be produced by using scalable and cost-effective techniques, making them a viable option for clinical translation. Recent studies also highlight the potential of NLC in enhancing the delivery of heat- or light-sensitive drugs, further broadening their applicability in complex dermatological conditions.^49,50^

Lipid-polymer hybrid nanoparticles combine the benefits of lipid-based carriers and polymeric systems, offering a versatile approach for drug delivery and creating an adaptable platform with enhanced structural integrity and functional capabilities. Depending on the desired properties, these systems consist of a lipid core surrounded by a polymer shell or vice versa. The hybrid design allows for precise customization of drug release profiles, enabling both immediate and sustained release as required by specific therapeutic goals^47^

For antimicrobial peptides, lipid-polymer hybrids offer unique advantages. The lipid component ensures biocompatibility and facilitates interaction with biological membranes, while the polymer shell provides protection against enzymatic degradation and enhances stability. These properties are particularly beneficial for AMP delivery, as they enable targeted action at the infection site while minimizing systemic exposure and potential cytotoxicity.^22,51,52^

Another significant advantage of lipid-polymer hybrids is their tunable size, charge, and surface properties, which can be optimized for specific applications. For instance, incorporating targeting ligands on the nanoparticle surface allows for selective delivery to infected tissues, improving the therapeutic efficacy. These systems can coencapsulate multiple agents, such as AMP and anti-inflammatory drugs, providing a synergistic approach to treating complex dermatological conditions.^53–55^

Although it shows highly promising attributes, there is a barrier intrinsic to encapsulating AMPs in lipid-based carriers, that is, the very nature of the AMP’s mechanism of action. The mechanism of action of many AMPs is cell lipid membrane disruption.^56,57^ While this is desirable at the target bacterial membrane, this same amphipathic and often cationic nature can compromise the structural integrity of the LNP carrier itself, potentially leading to premature drug release and reduced efficacy. Therefore, it is crucial to address this challenge when preparing the LNP formulation.

Hence, there are two primary strategies to effectively mitigate this AMP-induced destabilization. First, a well-established method is to incorporate cholesterol into the lipid matrix formulation, a well-established method to enhance lipid matrix stability. Cholesterol inserts itself between the phospholipid or lipid acyl chains, increasing their packing density and reducing membrane fluidity. This results in a more rigid and mechanically resilient bilayer that is significantly more resistant to peptide-induced disruption, pore formation, and leakage. By rigidifying the LNP structure, cholesterol helps preserve the payload until the nanoparticle reaches its target site.^58,59^

Another way to overcome this challenge is the inclusion of PEGylated lipids (lipids conjugated to polyethylene glycol). These molecules create a dense, hydrophilic polymer brush on the nanoparticle’s surface. This “steric shield” physically hinders the approach and interaction of the encapsulated AMP with the LNP’s outer surface, preventing the initiation of disruptive events. This strategy not only enhances stability against the payload but also improves colloidal stability and potentially prolongs circulation time in systemic applications.^60^

Despite their promising attributes, challenges such as scale-up production, cost considerations, and regulatory hurdles remain active research areas. Efforts to streamline manufacturing processes and develop standardized protocols are essential for advancing lipid-polymer hybrid nanoparticles toward clinical use.

### 3.1 Controlled Drug Release with Lipid Nanoparticles

A key benefit of using LNPs for drug delivery is their ability to release therapeutic agents in a controlled manner. This is particularly important for AMPs, as maintaining stable concentrations for extended periods enhances effectiveness while minimizing toxicity and reducing the chances of bacterial resistance.^25,34,47^

The use of LNPs as nanocarriers for AMPs provides a controlled drug release profile tailored to the target site. The physicochemical properties of LNPs, such as lipid composition and particle size, modulate drug release kinetics and ensure prolonged antimicrobial activity at infection sites. This controlled release not only improves therapeutic efficacy but also minimizes the risk of drug resistance by maintaining consistent drug levels over time.^11,13,14^

The controlled release of AMP from LNP is primarily governed by the physicochemical properties of the lipid matrix, the drug’s interaction with the lipid components, and the environmental conditions at the target site. For example, the crystallinity of SLNs often slows the diffusion of encapsulated drugs, providing a sustained release profile that can be particularly beneficial for chronic infections requiring prolonged treatment. Otherwise, NLCs, due to their less-ordered lipid matrix, offer more flexibility in modulating release rates, allowing for tailored drug delivery depending on the clinical requirements.^13,47,48^

One of the key benefits of LNPs is their ability to modulate the release of AMPs through the intrinsic properties of the lipid matrix. Studies indicate that passive and active delivery systems can be tailored to respond to local stimuli, such as pH and specific enzymes. This functionality enables encapsulated AMPs to target infection sites with higher precision, minimizing adverse effects and maximizing efficacy against resistant pathogens.^19,20,25^

LNP-mediated drug release occurs via several mechanisms (Figure 2), including diffusion, where the drug gradually diffuses from the lipid matrix to the surrounding environment. This process is influenced by the lipid composition and particle size, with smaller particles often facilitating faster release due to their larger surface area.^20,34,61^ Moreover, LNPs provide a microenvironment that protects AMPs against external stressors, such as enzymatic degradation, temperature, oxidation, and pH variation. Figure 2 illustrates how AMP-loaded LNPs shield the peptides from destabilizing factors and are released through multiple controlled mechanisms. These include passive diffusion, matrix erosion, and cellular uptake processes like endocytosis or membrane fusion, which ensure prolonged peptide activity and reduced systemic exposure.^14,20,34^

**Figure 2:**
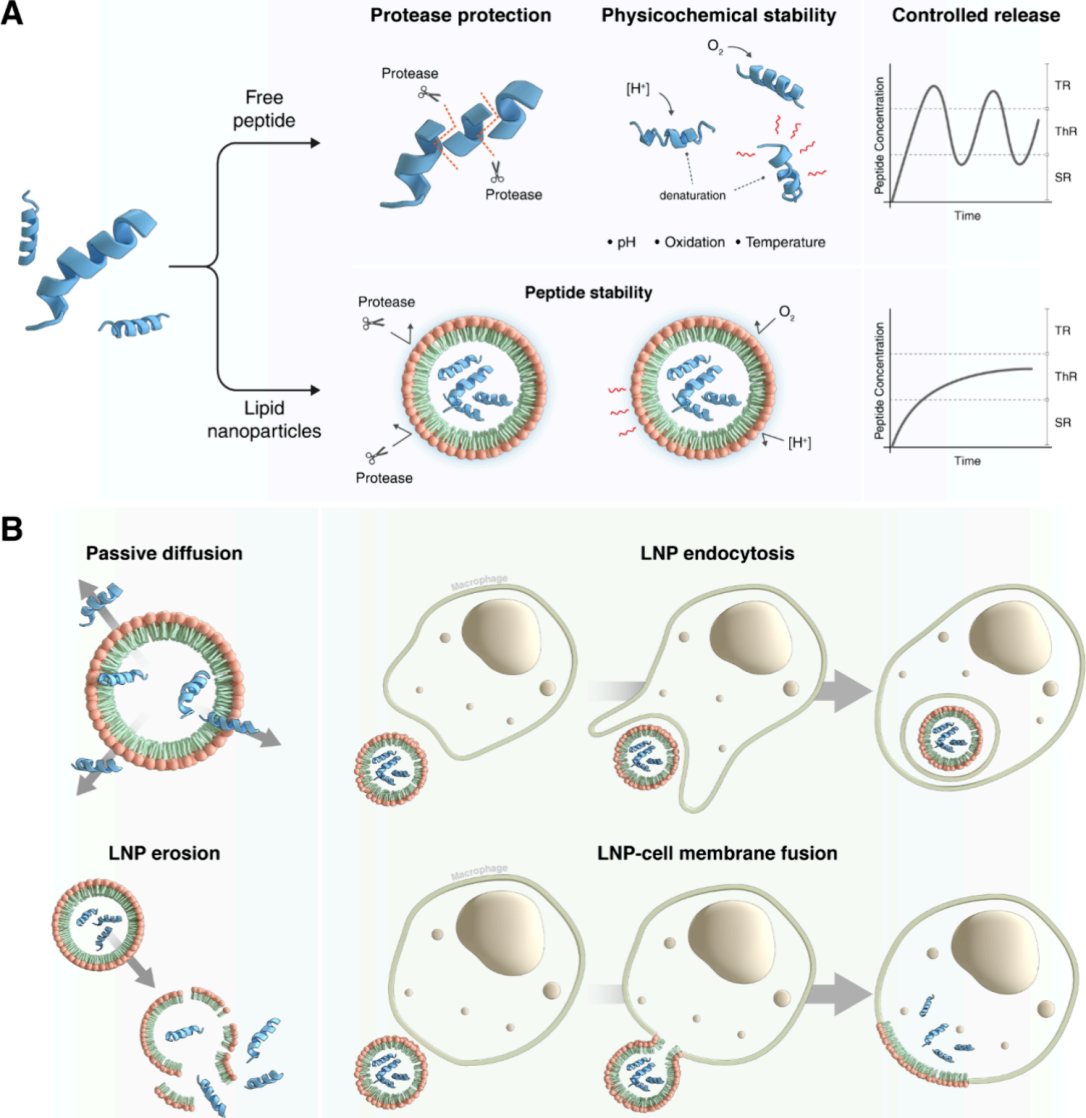
(A) Comparison between free and LNP-loaded peptides in terms of protection against proteolytic degradation, physicochemical stabilization (against pH, oxidation, and temperature), and ability to sustain and control release. (B) Potential release mechanisms of AMPs from LNPs, including passive diffusion, LNP erosion, endocytosis by phagocytic cells, and membrane fusion, enabling intracellular or extracellular peptide delivery. TR: Toxic range; ThR: Therapeutic Range; SR: Subtherapeutic range.

SLNs demonstrated a controlled release profile for lacticin 3147, extending antimicrobial activity to 11 days compared to 9 days for nonencapsulated formulations. This sustained release was attributed to the lipid matrix composition and nanosized particles, which enhanced peptide stability and bioavailability. Such features ensure prolonged contact with the infected site, effectively reducing bacterial colonization and facilitating wound healing.^62^

Another delivery possibility for the LNPs is erosion of the lipid matrix, in which the degradation of lipid components under physiological conditions can lead to the sustained release of encapsulated drugs. This mechanism is particularly relevant for biodegradable lipids, commonly used in LNP formulations.^20^

A biphasic release profile for LL-37 and serpin A1 encapsulated in SLNs, characterized by an initial burst release, followed by sustained delivery over 15 days. This controlled release pattern was attributed to the lipid matrix composition, which facilitated the slow diffusion of peptides while maintaining their bioactivity. Such release kinetics are critical for chronic wound management, where prolonged antimicrobial and anti-inflammatory effects, evinced by the attenuation of IL-1β, IL-6, and TNF-α levels, are necessary to prevent bacterial proliferation and promote tissue repair.^21^

Certain LNPs are designed to respond to specific stimuli, such as pH, temperature, or enzymatic activity, which present a delivery method called triggered release. pH-sensitive LNPs represent an advanced strategy for the targeted delivery of AMPs in environments with distinct pH variations, such as infected or inflamed tissues. The microenvironments of these sites often exhibit a lower pH due to the increased metabolic activity and localized immune responses. Leveraging this pH disparity, pH-sensitive LNP can enhance the therapeutic precision and efficacy of the entrapped active ingredient by ensuring release only at the target site, minimizing off-target effects and systemic toxicity, mainly due to maintaining the peptide for a long time inside the therapeutic range (Figure 2A).^20,63^

These systems are typically engineered by incorporating lipids or surfactants that undergo structural or compositional changes in response to acidic conditions. For example, pH-sensitive LNPs may include protonatable lipids or pH-responsive polymers, destabilizing the nanoparticle structure in acidic environments. This destabilization facilitates the release of encapsulated AMPs, ensuring that therapeutic concentrations are achieved precisely at the site of infection or inflammation^20,64,65^

It was demonstrated that NLCs significantly enhanced the therapeutic profile of LL37 by addressing its inherent limitations. Entrapment of the AMP within pH-sensitive LNP provided controlled and selective release, ensuring that the therapeutic agent was released only in acidic environments, such as those found in infected or inflamed tissues. This targeted delivery minimized off-target effects and reduced systemic toxicity, which is a common concern with free AMPs. Moreover, the protective environment of the LNP shielded the AMP from enzymatic degradation, preserving its bioactivity and extending its therapeutic half-life. These advancements not only improved the peptide’s efficacy but also reduced the risk of adverse side effects, highlighting the potential of LNP as a transformative platform for AMP-based therapies.^63^

Molecular dynamics simulations were previously performed, providing mechanistic insights into how SLNs enhance the antibacterial efficacy of nisin Z.^16^ The simulations revealed that SLNs facilitate the spatial confinement of nisin Z at the bacterial membrane interface, promoting more potent and longer-lasting interactions with lipid II, which is a key bacterial cell wall precursor. This synergistic interaction amplifies the peptide’s ability to disrupt bacterial membranes, highlighting a novel mechanism by which SLNs enhance antimicrobial activity.^16^

Controlled release is particularly advantageous in dermatological applications, where maintaining localized drug levels can reduce systemic side effects and enhance patient adherence. Encapsulation of AMPs in LNPs ensures that therapeutic concentrations are kept at the infection site, minimizing the need for frequent reapplication and reducing the risk of bacterial resistance.^20,23^

## 4 *In Vitro* and *In Vivo* Efficacy

*In vitro* studies are essential to assess the entrapment efficiency, stability, release profiles, and protective effects of LNPs against enzymatic degradation. These evaluations provide insights into how LNP formulations behave under conditions that mimic biological environments, which is critical for predicting their therapeutic potential in treating skin infections.^18,20^

The efficacy of LNPs extends beyond *in vitro* environments, with *in vivo* studies providing critical validation for their therapeutic potential. While *in vitro* assays focus on encapsulation efficiency, stability, and protection against enzymatic degradation, *in vivo* models evaluate the ability of LNPs to target infection sites and enhance wound healing. These complementary studies bridge the gap between formulation development and clinical application, offering a comprehensive understanding of LNP performance under biologically relevant conditions.^14,16^

The efficacy of nanostructured lipid carriers (NLC) encapsulating LL-37 was demonstrated through *in vitro* and *in vivo* studies. *In vitro* assays confirmed the maintenance of LL-37 bioactivity postencapsulation, showing significant inhibition of macrophage activation induced by lipopolysaccharide (LPS). The encapsulated peptide effectively neutralized inflammatory mediators, a key factor in chronic wound environments. *In vivo*, the NLC-LL37 system showed superior wound healing properties in a diabetic mouse model, achieving significant improvements in wound closure, re-epithelialization, and collagen deposition compared to free LL-37. These findings highlight the potential of NLC-LL37 as a biocompatible and efficient therapeutic option for managing chronic wounds.^22^

Entrapment efficiency (EE) is a key parameter in determining the effectiveness of LNPs in delivering AMPs. SLNs and NLCs demonstrated significant entrapment efficiencies for peptides when a hydrophobic ion pair (HIP) was formed. HIPs enhance the lipophilicity of hydrophilic peptides, which improves their compatibility with the lipid matrix. In that study, NLC achieved higher EE compared to SLN, with encapsulation efficiencies of up to 78% for HIP-encapsulated peptides.^66^ Table 2 provides an overview of the AMP-loaded LNP.

**Table 2:** Overview of Lipid Nanoparticles (LNP) Incorporating Antimicrobial Peptides: Types, Applications, and Key Findings

type of LNP	incorporated peptide	key findings	methodological limitations	reference
solid lipid nanoparticles (SLN)	lacticin 3147	enhanced antimicrobial activity, sustained release up to 11 days, and significant bacterial eradication in *ex vivo* models	incorporating preformed SLNs into a hydrogel matrix without causing nanoparticle aggregation or destabilization is a critical and challenging step	^62^
solid lipid nanoparticles (SLN)	lacticin 3147	superior antimicrobial activity, dual-occupancy SLNs optimized synergistic action of peptides, and adequate protection against enzymatic degradation in GI conditions	achieving a consistent and reproducible loading ratio for the two synergistic peptide components during fabrication is a significant challenge for scalability	^67^
solid lipid nanoparticles (SLN)	LL-37 and serpin A1	accelerated wound healing, synergistic antibacterial activity, and controlled peptide release over 15 days with low cytotoxicity	the preparation method must be optimized to efficiently load two distinct peptides with different physicochemical properties, which can be difficult to control and reproduce	^21^
solid lipid nanoparticle (SLN)	polymyxin B	high encapsulation efficiency (>90%), stable particle size (∼200 nm), and effective antimicrobial activity against *Pseudomonas aeruginosa*	hot homogenization is a common SLN preparation method, which can expose the peptide to thermal stress, potentially leading to degradation if not precisely controlled	^43^
nanostructured lipid carriers (NLC)	LL-37	improved wound healing, enhanced re-epithelialization, and reduced inflammation	NLCs are often produced using high-pressure homogenization or ultrasonication. These high-energy methods can induce mechanical stress on the peptide, risking its structural integrity	^22^
nanostructured lipid carriers (NLC)	nisin Z	reduced minimum inhibitory concentration by 4-fold, enhanced stability, and improved bacterial membrane disruption efficiency	the fabrication process (e.g., high-shear homogenization) must be carefully optimized to ensure the nisin peptide remains fully bioactive and is not denatured by mechanical stress	^16^
nanostructured lipid carriers (NLC)	nisin Z	enhanced antimicrobial activity against *Staphylococcus aureus* and *Enterococcus faecalis*; improved peptide stability	while NLCs have a high loading capacity, a key challenge in their preparation is preventing the expulsion of the encapsulated peptide from the lipid matrix during cooling and storage	^68^
pH-responsive LNP	LL-37	effective targeted release under acidic conditions, improved bioavailability, and superior bacterial clearance in chronic wound models	the formulation is based on a self-assembly process, which can be difficult to control and scale up for industrial production while maintaining consistent nanoparticle size and responsiveness	^63^

The potential of SLNs entrapping lactic 3147 for treating chronic wound infections caused by *S. aureus*, including MRSA, was studied. *In vitro*, the SLNs enhanced antibacterial activity, achieving complete bacterial inhibition at concentrations as low as 31.25 μg mL^–1^. *Ex vivo* experiments using pig skin models showed significant bacterial eradication (>75%) after 1 h of application. These findings highlight the ability of SLNs to sustain antimicrobial efficacy over prolonged periods, improving wound management strategies.^62^

Also, SLNs containing lacticin 3147 showed superior antimicrobial activity compared to SLNs encapsulating individual peptides. These SLNs achieved 99.9999% bacterial inhibition against *Listeria monocytogenes* at concentrations as low as 3.125 μg mL^–1^. The dual-occupancy design ensured the synchronized release of both peptides, optimizing their synergistic action and resulting in higher efficacy in bacterial eradication compared to single-occupancy SLNs.^67^

Protease degradation studies revealed that NLC provided superior protection against enzymatic activity compared to SLN, particularly against trypsin. This protection was attributed to the inclusion of liquid lipids in the NLC structure, which reduced the extent of protease penetration and hydrolysis within the nanoparticle core. Conversely, SLN offered minimal protection due to their more rigid crystalline structure, which failed to shield encapsulated peptides effectively.^12,14^

Also, it was proven that NLC entrapment of peptides, namely, LL37, protects against chemical degradation and the ones caused by proteases. Moreover, they also extend the duration of the effect of the peptides, which results in lowering the high dosing frequency.^22^

The versatility of lipid nanosystems was highlighted, enhancing the efficacy of antimicrobial peptides (AMP). *In vitro* studies demonstrated that encapsulation within LNP significantly increased the AMP stability by protecting it from enzymatic degradation and interaction with plasma proteins. Furthermore, *in vivo*, these nanosystems enabled sustained release and precise targeting of AMPs, reducing systemic toxicity and enhancing antimicrobial activity. This dual benefit underscores the potential of LNPs in translating AMP-based therapies into clinical practice.^11^

*In vitro* and *in vivo* studies have demonstrated that LNPs outperform other nanocarriers, such as liposomes and polymeric nanoparticles, in terms of stability and controlled release. For instance, NLCs reportedly exhibited superior entrapment efficiency and release kinetics compared to traditional liposomes, particularly under conditions mimicking inflamed skin. These findings highlight the unique advantages of LNP in enhancing AMP delivery while minimizing off-target effects.^14^

While numerous studies demonstrate the general success of AMP-LNP systems, a more critical analysis is needed to understand which specific formulation parameters have a strong influence on therapeutic efficacy. Factors such as particle size, surface charge (zeta potential), and composition of the lipid matrix are not incidental; they are key determinants of how the nanoparticle interacts with biological barriers and releases its payload. To elucidate these relationships, Table 3 provides a comparative analysis of representative studies, correlating their formulation parameters with *in vitro* and *in vivo*/*ex vivo* outcomes.

**Table 3:** Correlation of Antimicrobial Peptide (AMP)-Loaded Lipid Nanoparticle (LNP) Formulation Parameters with Antimicrobial and Wound-Healing Efficacy^a^

LNP formulation (AMP+carrier)	key formulation parameters	*in vitro* efficacy outcome	*in vivo*/*in(ex) vivo* efficacy outcome	structure–function correlation	reference
LL-37 in NLC	size: ∼230 nm; PDI: <0.2; zeta: ∼−25 mV	maintained the anti-inflammatory activity of LL-37 against LPS-stimulated macrophages	significantly improved wound closure and re-epithelialization in a diabetic mouse model compared to free LL-37	the NLC’s amorphous matrix was the key determinant, providing sustained release essential for long-term immunomodulation and wound healing	^22^
lacticin 3147 in SLN	size: ∼250 nm; PDI: <0.3; zeta: ∼−30 mV	MIC of 31.25 μg/mL against MRSA, demonstrating high antimicrobial potency	>75% bacterial eradication on an *ex vivo* porcine skin model after one hour	the nanosize and occlusive properties of the SLN hydrogel likely enhanced skin contact and peptide bioavailability at the infection site	^62^
nisin Z in SLN	size: ∼160 nm; PDI: <0.2; zeta: ∼+30 mV	4-fold reduction in MIC against *S. aureus*; enhanced membrane pore formation observed via microscopy	more efficient reduction of bacterial loads in an *in vivo* infection model compared to the free peptide	the positive zeta potential was a critical factor, enhancing electrostatic attraction to negatively charged bacterial membranes and maximizing the peptide’s disruptive mechanism	^16^
LL-37 & serpin A1 in SLN	size: ∼150 nm; PDI: <0.2; zeta: ∼−15 mV	accelerated wound closure in fibroblast and keratinocyte cell cultures	synergistic antibacterial activity against *S. aureus* and *E. coli*; reduced bacterial burden in a wound model	the small particle size facilitated cellular interactions, while the solid matrix provided a controlled corelease of the two synergistic peptides	^43^

a NLCs: Nanostructured lipid carriers; SLNs: Solid Lipid Nanoparticles; PDI: Polydispersity Index.

As the analysis in Table 3 suggests, a clear structure–function relationship governs the success of AMP-LNP therapies. The data collectively indicate that (1) a particle size below 300 nm is consistently associated with effective performance, likely by facilitating penetration through biological barriers like the stratum corneum and biofilm matrix; (2) a strong cationic surface charge can significantly enhance antimicrobial activity by promoting electrostatic interactions with bacterial membranes, as seen with the nisin Z-SLN system; and (3) the lipid matrix composition (crystalline SLN vs amorphous NLC) is the primary determinant of the drug release profile, with sustained-release systems being crucial for long-term effects like wound healing. These correlations are vital for the rational design of next-generation nanocarriers for skin infections^16,22,43,62^

NLCs and SLNs were extensively evaluated for their *in vitro* release profiles and occlusion properties. The use of Franz diffusion cells, using a nylon membrane, revealed that SLN formulations provided a slower release of active compounds than NLC due to their more rigid lipid matrix. This sustained release is advantageous for maintaining therapeutic levels over time in topical applications. Additionally, the occlusive capacity of the lipid-based systems demonstrated significant potential for enhancing skin hydration, which is critical for wound healing and reducing transepidermal water loss.^14^

SLNs significantly enhanced the antibacterial activity of an AMP, the nisin Z. The SLNs achieved a 4-fold reduction in the minimum inhibitory concentration against *Staphylococcus aureus* compared to free nisin Z. The study revealed that SLN encapsulation improved the peptide’s stability and amplified its membrane-damaging mechanism of action. Scanning and transmission electron microscopy showed that nisin Z-loaded SLNs produced more prominent and more numerous pores in bacterial membranes, accelerating cell death within 30 min at a fraction of the free peptide’s concentration.^16^

The enhanced therapeutic potential of a combination solid lipid nanoparticle (SLN) formulation encapsulating LL-37 and serpin A1 was previously demonstrated. *In vitro* studies showed that this formulation accelerated wound healing in BJ fibroblast cells and keratinocytes, significantly improving wound closure compared with free LL-37 or A1. *In vivo*, the SLN system exhibited synergistic antibacterial activity against *S. aureus* and *E. coli*, reducing the bacterial burden and promoting tissue regeneration in chronic wound models. These results underline the importance of SLNs in maintaining peptide stability and achieving controlled release, which is essential for sustained antimicrobial and wound-healing effects.^21^

*In vivo* studies highlighted the superior performance of nisin Z-loaded SLNs in achieving rapid bacterial death. Compared to free nisin Z, the SLN formulation reduced bacterial loads more efficiently, requiring significantly lower AMP concentrations to achieve bactericidal effects. These findings underscore the potential of SLNs to enhance the therapeutic index of antimicrobial peptides, making them viable candidates for clinical applications targeting multidrug-resistant infections.^16^

A primary advantage of LNP entrapment is the substantial protection it offers against enzymatic degradation, a major factor limiting the *in vivo* efficacy of free peptides.^34^ The solid lipid matrix acts as a physical shield, sterically hindering access to proteases prevalent in biological fluids. This has been quantitatively demonstrated in several studies. For example, in experiments simulating gastrointestinal conditions, SLNs were shown to effectively protect the bacteriocin lacticin 3147 from digestive enzymes like pepsin and pancreatin, preserving its antimicrobial activity, whereas the free bacteriocin was rapidly inactivated.^31,62,69^ Another comparative study found that nanostructured lipid carriers (NLCs) provided superior protection for encapsulated model peptides against trypsin, with less than 20% of the peptide being degraded after two hours of exposure. In stark contrast, the free, unencapsulated peptide was almost completely degraded (>90%) within the same time frame. This dramatic increase in stability directly translates to a prolonged therapeutic half-life, ensuring the peptide remains bioactive and available at the target site for an extended period.^66^

Incorporating bacteriocins into lipid nanoparticles (LNP) represents a promising strategy to overcome their inherent limitations. The potency of AMPs at low concentrations against multidrug-resistant species was demonstrated, but their low *in vivo* stability remains challenging. The protective environment provided by LNP can shield bacteriocins from enzymatic degradation, maintaining their activity in systemic infections and localized treatments.^3^

One of the most significant challenges in treating chronic skin infections is the formation of bacterial biofilms.^70^ These complex communities are encased in a self-produced extracellular polymeric substance (EPS) matrix, which acts as a physical barrier, protecting the embedded bacteria from both host immune responses and conventional antibiotics. While AMPs possess intrinsic antibiofilm properties, their efficacy can be limited by poor penetration into the dense EPS matrix and degradation by bacterial proteases concentrated within the biofilm.^70,71^

Lipid nanoparticles offer a strategic solution to overcoming these barriers. Due to their small size and lipophilic surface, LNPs can more effectively penetrate the channels and pores within the biofilm structure.^72^ Furthermore, they can protect the encapsulated AMP from enzymatic degradation, acting as nanoscopic depots that provide a sustained release of the peptide directly inside the biofilm. This ensures that a high, localized concentration of the AMP is maintained over time, which is critical for eradicating the slow-growing and persistent bacteria within the community. Several studies have validated this hypothesis, demonstrating that encapsulating AMPs within LNPs significantly enhances their antibiofilm potency.^13,31,72^

Previous studies demonstrate consistent data which show that LNP-based delivery systems outperform free AMPs in both preventing the formation of and eradicating established biofilms.^73–75^ The sustained release profile offered by LNPs^61,76^ is particularly advantageous, as it maintains a high local peptide concentration within the biofilm matrix, overcoming the tolerance often observed in these bacterial populations. This enhanced antibiofilm activity is critically important for the potential treatment of persistent, nonhealing conditions, such as diabetic foot ulcers or chronic wound infections, where biofilms are a primary cause of therapeutic failure.^73,74^

Beyond biofilms, another significant challenge in treating skin infections is the ability of certain pathogens, notably *Staphylococcus aureus*, to invade and survive within host cells, such as keratinocytes and macrophages. This intracellular reservoir protects the bacteria from many antibiotics and free AMPs, which typically exhibit poor cell penetration, leading to persistent or recurrent infections.^77^

In this context, LNPs can be significantly more efficient than free AMPs by acting as a “Trojan horse”.^78^ Nanoparticles, particularly those with specific surface properties (e.g., a cationic charge), can be actively taken up by host cells through endocytosis. Once internalized, the LNP can deliver its AMP payload directly into the intracellular compartments, where the bacteria reside. This targeted intracellular delivery allows AMP to eradicate the hidden bacterial reservoir, an outcome that is often unachievable with the free peptide alone. This ability to target and eliminate intracellular pathogens represents a key advantage of LNP-based delivery, enhancing its therapeutic potential for treating persistent and difficult-to-cure skin infections^13,14,16^

## 5 Toxicity and Bioavailability

The successful clinical application of LNPs relies heavily on balancing maximizing bioavailability and minimizing toxicity. These factors are interdependent, as increased bioavailability of AMPs must be achieved without compromising patient safety or causing adverse effects.

LNP systems generally enhance the bioavailability of AMPs primarily by protecting them from enzymatic degradation and facilitating their transport to the target site. One of the main barriers of AMPs is their short elimination half-life, which results from their fast proteolytic degradation. Once in the bloodstream, the lipid matrix acts as a barrier against proteolytic enzymes, significantly prolonging the half-life of AMPs under physiological conditions. Additionally, the ability of LNPs to merge with the lipid bilayers of the stratum corneum improves skin penetration, ensuring that AMPs reach deeper layers where pathogens often reside.^33,79^

Controlled release mechanisms (Figure 2), including those mediated by pH-sensitive or enzyme-responsive LNPs, enhance biodistribution by ensuring targeted delivery of AMP to infected tissues while minimizing systemic exposure. This targeted delivery not only enhances therapeutic efficacy but also reduces the required dosage, thereby improving patient compliance.^19,20^

LNPs significantly enhance the bioavailability of antimicrobial peptides (AMPs) by protecting them from enzymatic degradation and facilitating their delivery to target sites. For example, NLC formulations improve the stability of hydrophilic AMPs by incorporating them into a lipid matrix that shields the peptides from proteolytic enzymes. Studies show that AMP-loaded LNP achieve sustained release over extended periods, maintaining therapeutic concentrations while minimizing the frequency of application.^66^

While LNPs help stabilize AMPs and improve their absorption, their formulation must be carefully designed to ensure they remain safe for use. Using biocompatible lipids and mild surfactants has helped reduce toxicity concerns, and newer approaches, such as pH-sensitive nanoparticles, are making drug delivery more precise, minimizing unwanted side effects.^67^

Additionally, pH-sensitive LNP systems improve bioavailability by selectively releasing their payload in acidic microenvironments such as infected tissues. It was demonstrated that LNP loaded with nisin Z achieved 10-fold higher local concentrations in preclinical infection models compared to free AMP, highlighting their efficacy in enhancing drug localization and reducing systemic exposure.^16^

The lipids commonly used to formulate LNPs, such as triglycerides, fatty acids, and phospholipids, are generally considered highly biocompatible and nonimmunogenic, as they are endogenous to the body. However, the surfactants required to stabilize the LNP dispersion can, at certain concentrations, cause mild skin irritation. Nevertheless, numerous preclinical *in vivo* studies have demonstrated excellent overall skin tolerance for LNP formulations, with minimal to no signs of erythema or edema upon application to intact or wounded skin. While these preclinical data are very promising, comprehensive dermatological safety testing in humans, including patch tests for sensitization, remains a crucial step for clinical translation. Hence, lipid type, surfactant concentration, and drug loading can influence the cytotoxicity. However, it has already been shown that SLN formulations had lower cytotoxic effects than free compounds in cell viability assays, likely due to controlled release mechanisms that prevent peptide overexposure.^13,14^

SLNs entrapping the dual-acting bacteriocin lacticin 3147 effectively protected the peptide from enzymatic degradation under simulated gastrointestinal conditions. Encapsulation enhanced the peptide’s bioavailability and maintained its antimicrobial efficacy during transit through the gastrointestinal tract. This finding underscores the critical role of SLNs in preserving the bioactivity of peptides in harsh biological environments, which is essential for achieving therapeutic outcomes while minimizing systemic exposure.^67^

The NLC-LL37 formulation demonstrated high biocompatibility with no significant cytotoxic effects observed in human fibroblast cell lines at therapeutic concentrations. The entrapment within NLC protected against proteolytic degradation, enhancing the bioavailability of LL-37 at the target site. This sustained release profile reduced the need for frequent dosing, offering a practical advantage for chronic wound management. Furthermore, the topical application of NLC-LL37 minimized systemic exposure, aligning with safety requirements for long-term therapies.^22^

The SLN formulation of LL-37 and serpin A1 demonstrated low cytotoxicity in BJ fibroblast cells and keratinocytes at therapeutic doses. Encapsulation within SLNs protected the peptides from degradation and aggregation, enhancing their bioavailability at the wound site. This protective effect extended the therapeutic window of both peptides, ensuring steady release over 15 days. Furthermore, the SLNs minimized systemic exposure, reducing potential side effects and aligning with the safety requirements for chronic wound therapies.^22^

The clinical potential of bacteriocins is often constrained by their inherent instability and vulnerability to proteolytic enzymes *in vivo*. These peptides are rapidly degraded in the systemic circulation, limiting their therapeutic efficacy and requiring frequent or high-dose administrations, which could lead to off-target effects. LNPs offer a promising solution to this challenge by encapsulating bacteriocins within a protective lipid matrix. This encapsulation shields the peptides from enzymatic degradation and enhances their plasma half-life and site-specific delivery. Recent studies demonstrate that LNPs preserve the bioactivity of entrapped AMPs and minimize systemic toxicity by ensuring targeted delivery and controlled release, as shown in preclinical studies, paving the way for their broader application in clinical settings.^3,11^

Bacteriocins have demonstrated significant potential as antimicrobial agents due to their selectivity and low toxicity. However, their bioavailability is limited by rapid enzymatic degradation and poor systemic stability. LNP formulations offer a viable solution, enhancing the metabolic stability and plasma half-life of these peptides while minimizing off-target effects.^3^

A critical aspect of leveraging LNPs for AMP delivery is their profound impact on the therapeutic index, that is, maximizing antimicrobial efficacy while minimizing host cell toxicity. A primary obstacle for the clinical translation of many potent AMPs is their potential for off-target effects, including cytotoxicity against human cells (e.g., keratinocytes and fibroblasts) and hemolytic activity, often due to the same membrane-disrupting mechanisms that kill pathogens.

LNP encapsulation directly mitigates this issue by modulating the peptide’s interaction with host tissues. By sequestering AMP within a lipid matrix, the delivery system prevents high concentrations of the free peptide from coming into direct contact with host cell membranes. Instead, a controlled and sustained release maintains the local concentration within a therapeutic window that is effective against bacteria but remains below the threshold for host cell toxicity.^80^

This improved safety profile has been consistently demonstrated both *in vitro* and *in vivo*. For example, a previous study showed that NLCs loaded with the peptide LL-37 exhibited no significant cytotoxic effects on human fibroblast cell lines at therapeutic concentrations, showcasing a high degree of biocompatibility.^22^ This is a considerable improvement, as free LL-37 can induce cytotoxic effects at higher doses. Similarly, SLN formulations encapsulating LL-37 and serpin A1 were found to have a low cytotoxicity in both fibroblast and keratinocyte cell lines. This principle is especially valuable for potent but historically toxic peptides.^21^ For instance, new formulations of Polymyxin B encapsulated in LNPs have been shown to significantly reduce its nephrotoxicity and hemolytic activity compared to the free drug, potentially enabling the safer systemic use of this last-resort antibiotic.^43^

In summary, the LNP platform not only enhances the stability and bioavailability of AMPs but also fundamentally improves their safety profile, representing a crucial step toward the clinical viability of these powerful antimicrobial agents.

However, it was noted that potential inflammatory responses were associated with high nanoparticle concentrations, emphasizing the importance of dose optimization. Future research should focus on refining lipid compositions and developing nontoxic surfactants to ensure the safety of LNP formulations during long-term use.^16^

## 6 Challenges and Future Directions

While the synergy between AMPs and LNPs presents a transformative approach for treating skin infections, the path from promising preclinical findings to widespread clinical use is paved with significant challenges. Overcoming these hurdles will require a concerted interdisciplinary effort focused on formulation optimization, manufacturing scalability, and rigorous safety evaluation.

The next generation of AMP-LNP systems will likely move beyond simple encapsulation. A key future direction is the development of stimuli-responsive nanoparticles that release their payload in response to specific triggers in the microenvironment of an infection. This can be achieved by incorporating lipids with pH-sensitive linkers that cleave in the acidic environment of infected tissue, or by designing the LNP matrix to be degraded by bacteria-specific enzymes, ensuring a highly targeted release of the AMP.^81,82^

Furthermore, creating hybrid nanosystems that combine the benefits of LNPs with those of other materials, such as polymers or hydrogels, could enhance stability and improve skin adhesion. The potential for coencapsulating AMPs with synergistic antibiotics to overcome resistance, or with anti-inflammatory agents to modulate the wound environment, represents a powerful strategy for creating multipronged therapeutic systems that can address the complex pathophysiology of chronic wounds.^10,19,67,69^

Despite the wealth of promising preclinical data, the successful translation of AMP-LNP formulations from the laboratory bench to clinical application is a significant undertaking that hinges on surmounting key manufacturing and regulatory challenges. The path to a commercially viable and clinically approved product is complex, requiring robust, scalable, and reproducible production processes governed by stringent quality control.^82^

A primary obstacle is the scalability of the manufacturing. Many common lab-scale fabrication methods, such as probe sonication, are challenging to scale up and often lead to significant batch-to-batch variability. For industrial production, methods like high-pressure homogenization (HPH) and, more recently, microfluidics are far more suitable.^83,84^

Beyond the technical challenges of scalability, the economic feasibility of production is a critical determinant for the commercial deployment of AMP-LNP therapies.^35^ The primary cost drivers include the GMP-grade synthesis of the therapeutic peptide and the procurement of highly pure pharmaceutical-grade lipids. To ensure commercial viability, exploration of cost-effective and scalable manufacturing techniques is essential. For instance, continuous manufacturing is emerging as a promising strategy. Compared to traditional batch processing, continuous-flow systems can reduce operational costs, minimize facility footprints, and improve product consistency, thereby lowering the cost of goods. Optimizing formulation parameters, such as maximizing encapsulation efficiency to avoid wasting the expensive peptide payload, is also a key economic consideration. Developing these cost-effective strategies is paramount to ensuring that the final therapeutic product is not only effective but also accessible to patients.^16,35,62^

This leads directly to the need for rigorous Quality Control (QC) and the establishment of Critical Quality Attributes (CQAs). For any AMP-LNP formulation to gain regulatory approval under Good Manufacturing Practices (GMP), manufacturers must demonstrate the consistent production of a product with a predefined set of specifications, including particle size, encapsulation efficiency, peptide integrity, and long-term stability.

Addressing these challenges is essential for determining the real-world clinical path. The journey is undoubtedly challenging but becoming increasingly feasible. The monumental success of LNP-based mRNA vaccines has dramatically accelerated the field, forging clearer regulatory pathways with agencies such as the FDA and EMA. These precedents provide a solid foundation, making the clinical translation of AMP-LNP therapies a realistic yet demanding long-term goal.

These formulations also pose unique regulatory challenges, as they are often classified as combination products (biologic + drug delivery system), requiring a complex characterization and safety assessment strategy to satisfy regulatory agencies.

While the lipids used in LNP formulations are generally regarded as safe, the long-term safety of nanoparticles applied repeatedly to skin, especially compromised or wounded skin, remains underexplored and represents a critical knowledge gap. Future research must move beyond acute toxicity assays and incorporate chronic preclinical models to rigorously evaluate long-term safety. This should involve repeated topical application studies in animals with skin physiology similar to that of humans (e.g., porcine models) to assess potential tissue accumulation. Using LNPs with fluorescent or radiolabeled lipids in these models can help track biodistribution and determine whether nanoparticles are cleared or accumulate in the skin or distal organs over time.

To evaluate potential immune interactions, specific biomarkers should be monitored. Locally, this includes histological analysis of skin biopsies for signs of chronic inflammation and quantification of the expression of proinflammatory cytokines, such as TNF-α, IL-6, and IL-1β. Systemically, blood panels should be analyzed to detect subtle long-term inflammatory responses or signs of organ toxicity. A comprehensive understanding of these long-term interactions is essential for ensuring the safety of AMP-LNP therapies before they can be widely adopted in clinical practice^16^

Standardized methods for evaluating the LNP stability over time are also essential. Future research should focus on optimizing manufacturing processes and developing hybrid delivery systems that combine multiple strategies to enhance the therapeutic effects of AMPs.^19^

Future directions include developing multistimuli-responsive LNP systems that combine pH, temperature, and enzymatic triggers for precise drug delivery. Emerging techniques in lipid engineering and hybrid nanoparticle design also offer opportunities to enhance the efficacy and versatility of the LNP for complex skin infections and beyond.

## 7 Conclusions

Lipid nanoparticles (LNPs) have revolutionized drug delivery, offering solutions to long-standing challenges in antimicrobial peptide (AMP) therapeutics. By enhancing stability, enabling controlled release, and ensuring targeted delivery, LNPs are paving the way for the next generation of treatments against multidrug-resistant pathogens and chronic skin infections.

The fusion of nanotechnology and peptide-based therapies represents a significant leap forward, transforming AMPs from experimental agents to clinically viable solutions. These innovations address urgent global health challenges and redefine the standards of precision medicine, offering safer and more effective therapeutic options.

Furthermore, the principles outlined in this review are not limited to dermatology. The versatility of LNP systems extends their potential to treat affections in other challenging environments. This includes oral formulations with protective coatings for targeting gut pathogens, nebulized LNP systems for treating lung infections, such as those in cystic fibrosis, and surface-modified “stealth” LNPs for intravenous administration to combat systemic, biofilm-related infections.

Finally, while the preclinical findings discussed in this Review are crucial for establishing a proof of concept, it is imperative to acknowledge their limitations. Animal models, however sophisticated, cannot fully replicate the complex pathophysiology of chronic human skin infections or the nuances of the human immune response. Therefore, the ultimate validation of the safety and efficacy of AMP-LNP therapies can be achieved only through rigorously designed human clinical trials. These trials will be essential for comprehensively assessing not only therapeutic outcomes but also potential immune responses, the long-term risk of bacterial resistance development, and overall patient safety with repeated application. Bridging this critical translational gap from preclinical models to human studies represents the most significant and necessary next step in bringing these promising nanotherapies to patients who need them.

The path forward will undoubtedly require close collaboration among nanotechnologists, microbiologists, dermatologists, and regulatory scientists to bridge the gap between these promising innovations and tangible clinical solutions. With these in place, the battle against resistant infections can shift decisively, making personalized and accessible treatments a reality for patients worldwide.

Furthermore, the principles of stability and controlled release outlined in this review are not limited to dermatology. The versatility of LNP systems can be adapted to treat infections in other challenging environments. This includes oral formulations with protective coatings for targeting gut pathogens, nebulized LNP systems for treating lung infections such as those in cystic fibrosis, and surface-modified “stealth” LNPs for intravenous administration to combat systemic, biofilm-related infections, extending the platform’s potential far beyond the skin.
